# Cut Left or Left Alone, What to Do in the Middle of a Storm!

**DOI:** 10.1016/j.cjcpc.2022.11.006

**Published:** 2022-12-01

**Authors:** Joseph Atallah

**Affiliations:** Department of Pediatrics, Stollery Children’s Hospital, University of Alberta, Edmonton, Alberta, Canada

It is thought that the first description of left cardiac sympathetic denervation (LCSD) dates to 1899. It was suggested as a potential treatment for angina, and when applied several years later, serendipitous arrhythmia suppression was observed.^1^ Several decades later, in 1971, the first report of LCSD in long QT syndrome (LQTS) emerged.^2^ By the early 1990s, the value of LCSD in LQTS was affirmed.^3^ The antiarrhythmic impact of LCSD has since undergone a “renaissance.” It is now well accepted and adapted as an effective modality in the therapeutic armamentarium for inherited arrhythmias such as LQTS and catecholaminergic polymorphic ventricular tachycardia (VT).^4^ Although LCSD can serve the purpose of primary therapy in the rare circumstance of medication intolerance/contraindication, it is mainly an adjunctive treatment. It is specifically recommended in patients with high-risk phenotypes and breakthrough arrhythmic events on optimal medical therapy or when an implantable cardioverter defibrillator is contraindicated or refused. It is usually performed via an open surgical approach or using a thoracoscopic technique.^5^

In the article “Left cardiac sympathetic denervation as an acute treatment of torsades in a pediatric case of long QT,” ^6^ Shortland et al. describe the use of LCSD as an acute treatment in a patient with recalcitrant VT and suspected LQTS. The LCSD was concomitant with a transvenous implantable cardioverter defibrillator implantation within less than 1 week of initial presentation with recurrent VT and Torsade de Pointes. The patient demonstrated a favourable response with complete remission from ventricular arrhythmias and de-escalation of medical therapy, before home discharge. This scenario represents several layers of rare circumstances including the unusual clinical presentation and the resistance to antiarrhythmic drug therapy. LCSD is a rare procedure in general and its use as an acute therapy for the treatment of electrical storms is another rare occurrence that is not systematically described in the literature. However, the management of patients, especially of the paediatric age group, presenting in electrical storms can prove very humbling. When escalating antiarrhythmic drug therapy becomes a building block of failed attempts and resorting to extracorporeal membrane oxygenation is looming in the not so far horizon, another tool in the box is always desperately needed.

In this same daunting scenario of drug refractory ventricular tachyarrhythmia, percutaneous stellate ganglion nerve block has been described as an effective acute therapy in adult and paediatric patients with inherited arrhythmia.^7^^,^^8^ Although a temporary therapy, this less invasive approach can be done under fluoroscopic or ultrasound guidance and may provide insight into the efficacy of the more permanent and invasive surgical or thoracoscopic LCSD. Although this correlation has not been studied and proven, quieting the storm with percutaneous nerve block can be the stepping stone needed to leap from temporary to permanent LCSD and can provide a window of clinical stability to do so. Another percutaneous technique described in the literature is thoracic epidural anaesthesia (TEA). In a small series evaluating the antiarrhythmic effect of TEA, the response to deep sedation with suppression of ventricular arrhythmia was observed to be predictive of the response to TEA.^9^

It is important to note that although LCSD is effective in a large proportion of patients, some may have a suboptimal response with breakthrough arrhythmias and others may be nonresponders.^10^^,^^11^ Evidently, the underlying diagnosis and arrhythmia phenotype is one of the main confounders impacting procedural effectiveness. However, variation in the surgical technique and underlying left sympathetic chain anatomy is another important factor associated with the antiarrhythmic effectiveness of this procedure. Ventricular myocardial innervation overlap between the right and left stellate ganglia has been demonstrated.^12^ In addition, although LCSD is a preganglionic nerve interruption where reinnervation is not expected, data suggest that such may still occur.^13^ Therefore, resorting to a right sympathetic denervation for nonresponders is not always the right option. Revising the LCSD may be the better approach in some patients.

Cardiac myocardial nuclear imaging using 123-iodine metaiodobenzylguanidine (MIBG), a norepinephrine analog, can provide an objective assessment of the effectiveness of an LCSD. MIBG imaging performed before and after LCSD can prove helpful in differentiating between patients who are nonresponders due to residual myocardial sympathetic innervation and those in whom sympathetic stimulation is not a primary arrhythmia trigger. MIBG can also help identify patients whose arrhythmia relapse is due to reinnervation.^14^

Finally, when LCSD is used in young patients, it is important to recognize the possible presence of an age-dependent response. A variable effect on ventricular fibrillation threshold in maturing hearts has been identified in animal models but has not been studied in humans.^15^
